# Animal diseases and human future

**DOI:** 10.1186/s44149-022-00041-z

**Published:** 2022-04-25

**Authors:** Min Cui, Bang Shen, Zhen F. Fu, Huanchun Chen

**Affiliations:** 1 State Key Laboratory of Agricultural Microbiology, College of Veterinary Medicine, Huazhong Agricultural University, Wuhan, 430070 Hubei China; 2 Department of Pathology, College of Veterinary Medicine, University of Georgia, Athens, GA 30602 USA

Animals play an important role in our lives. Increased human-animal interactions lead to the transmission of zoonotic pathogens between animals and humans, directly threatening human health and societal development. Nearly all major public health events that occurred in the last several decades have been closely related to animal diseases. Approximately, 60% of human infectious diseases are zoonotic, and 75% of emerging human infectious diseases originate from animals. As of April 20, 2022, the global coronavirus infectious disease-19 (COVID-19) pandemic caused more than 503 million infections and more than 6.2 million deaths in humans. Over 11 billion doses of vaccines have been given to humans worldwide (WHO 2022). Despite the consensus among international organizations and animal experts that the SARS-CoV-2 originated from animals, the route of transmission between humans and other animals remains unclear. Furthermore, it is still a challenge for continually tracing the origin of SARS-CoV-2.

Animal diseases impose roughly 20% economic loss to the global animal industry every year. Emerging and re-emerging animal diseases also pose great challenges for the world. It has been more than 100 years since African swine fever (ASF) was first reported in Kenya in 1921. Due to the lack of effective vaccines and medications, ASF has devastated the swine industry in Africa. Subsequently, it has spread worldwide, causing considerable economic losses to the swine industry (Galindo and Alonso 2017).

In the meantime, frequent occurrences of animal diseases have led to the widespread antibiotic misuse and abuse. Every year, most antibiotics consumed are used for food production animals (Van Boeckel et al. 2015), resulting in an alarming increase of bacterial resistance and drug residues. It could potentially cause the emergence of super drug-resistant bacteria (superbugs). O’Neill (2015) predicted that “if the development trend of bacterial drug-resistance cannot be effectively curbed, approximately 10 million people will be killed every year by 2050, which will exceed the number of annual cancer deaths”.

Therefore, prevention and control of animal diseases and zoonoses not only meet the urgent needs of animal welfare and sustainable development of animal breeding industry, but also benefit human health and societal stability. More attention should be paid to the following three aspects with the challenges we face and the opportunities brought about by technology innovation.

1. We should establish research in etiology, epidemiology, and origin tracing of emerging and reemerging pathogens, which would help us better understand the origination, evolution, and spread of infectious pathogens as well as their pathogenesis and immune mechanisms, to design and implement superior strategies for control and prevention of animal diseases and zoonosis.
2. We should speed up the development of diagnostic reagents, new drugs, and novel vaccines to maximize the efficiency in prevention, control, and even elimination of infectious diseases.
3. We should integrate new technologies, such as synthetic biology and CRISPR gene editing, into animal disease control and disease-resistant animal breeding to facilitate the development of new products and novel control strategies.

There is no doubt that scientific and technological innovation is the driving force to improve animal health. Continuous, systematic, and sustainable research is absolutely necessary to effectively control and prevent animal diseases, to support the rapid development of the global veterinary medicine, and to eventually realize the vision of “One World, One Health.”
